# Hepatic changes by benznidazole in a specific treatment for Chagas disease

**DOI:** 10.1371/journal.pone.0200707

**Published:** 2018-07-20

**Authors:** Tycha Bianca Sabaini Pavan, Jamiro Wanderley da Silva, Luiz Cláudio Martins, Sandra Cecília Botelho Costa, Eros Antônio de Almeida

**Affiliations:** The Grupo de Estudo em doença de Chagas [GEDoCh/Unicamp], Department of Medical Clinic, Faculty of Medical Sciences, State University of Campinas (Unicamp), Campinas, São Paulo, Brazil; University of Navarra School of Medicine and Center for Applied Medical Research (CIMA), SPAIN

## Abstract

Chagas disease (Cd) is the third most common parasitic disease that causes damage to human health. Even a century after its description by Carlos Chagas and advances in its control, it remains a neglected disease. To eradicate the parasite or reduce the parasitic load, specific treatment for *Trypanosoma cruzi* (*T*. *cruzi*) is advisable; benznidazole (BNZ) is the drug that is currently prescribed. The purpose of this study is to report the adverse events (AE) due to the use of BNZ as a specific treatment for Cd, with a particular focus on hepatic changes. This was an observational, cross-sectional cohort study that included patients who were treated with BNZ. The medical records of patients who joined the Grupo de Estudo em doença de Chagas [Chagas Disease Study Group]/UNICAMP/Brazil and were treated with BNZ were reviewed for epidemiological, clinical, laboratory and AE parameters for the drug. The 204 patients who were assessed had an average age of 40.6 years ± 13.5 years, and 104 of them were women (50.98%). Fourteen (6.86%) individuals were in the acute phase of Cd, and 190 (93.13%) were in its chronic phase. AEs occurred in 85 patients (41.66%), 35 (41.17%) of whom had AEs related to the liver, characterized by an elevation of AST liver enzymes, ALT, alkaline phosphatase and gamma-glutamyltransferase (γGT). Other AEs that were observed included the following: 48 cases of cutaneous changes (56.47%), 8 cases of epigastric pain (9.41%), 7 cases of blood alteration (8.23%), and 3 cases of peripheral neuropathy (3.52%). Treatment was interrupted in 32 patients (37.64%) due to AD. Adverse events related to the liver secondary to the use of BNZ for Cd-specific treatment were frequent in this study and were characterized by an elevation of liver enzymes. Therefore, it is suggested that these enzymes be monitored during treatment with benznidazole.

## Introduction

Cd is caused by *T*. *cruzi* and is the third most common parasitic disease in the world after malaria and schistosomiasis [1]. It is classified as a neglected disease by the World Health Organization (WHO) [2]. Unfortunately, a century after its discovery, Cd still affects millions of people in Latin America and is one of the major causes of sudden death, arrhythmias, and heart failure [3,4].

Cd does not resolve spontaneously, and there is no effective treatment for the disease. However, two drugs have been indicated for its treatment: nifurtimox and benznidazole (BNZ) [5]. BNZ is the treatment of choice given its better treatment efficacy and fewer AEs [6]. BNZ is a nitroimidazole derivative that acts by interrupting protein synthesis, which impairs the formation of the parasite [7]. This drug is metabolized in the liver and may cause hepatic toxicity, which has been observed in experiments in which infected mice have elevated liver enzyme levels in the first 15 days of treatment with BNZ. Because there are few studies on the relationships among the parasitic infection, the drug and AEs related to the liver, there is a need for the monitoring of possible hepatic changes during treatment [6,8].

The use of drug in the acute phase can eradicate the parasite, which prevents progression to the chronic phase [9]. Treatment with BNZ is recommended to decrease parasitemia for patients with cardiopathy of a low degree of severity, which may consequently limit the clinical evolution of Cd [10]. Approximately 30% of adult individuals using BNZ experience AEs, such as a cutaneous reaction, epigastric pain, anorexia, neutropenia, elevations of creatinine and liver enzymes, and peripheral neuropathy. Because of these AEs, more than 20% of patients discontinue the treatment [9,11].

The potentiality of AEs related to the liver resulting from BNZ is unquestionable since its main metabolic mechanism is mediated by the cytochrome P450 enzyme complex. The biotransformation of the drug takes place in the hepatic tissue, with subsequent elimination by the kidneys [12]. These hepatic changes are less frequent than other AEs and may have a greater severity than what is reported, and their possible underdiagnosis motivated the present study [13]. Thus, the purpose of this study was to assess the presence and possible severities of hepatic AEs through clinical presentation and laboratory abnormalities, which were reviewed in chagasic patients treated with BNZ in the reference center.

## Methods

This was a retrospective, observational, cross-sectional cohort study conducted with the Grupo de Estudo em Doença de Chagas [Chagas Disease Study Group] (GEDoCh) at the State University of Campinas (Unicamp) in São Paulo, Brazil, a reference center in the care for individuals with Cd. The casuistry included 204 patients who started the treatment with BNZ; 104 patients (50.98%) were female and 100 were male (49.01%), and the minimum age and maximum age were three months and 78 years, respectively (40.6 years ± 13.5 years).

The treatment inclusion criteria were (1) to present two confirmatory serological tests of a Cd diagnosis according to the World Health Organization; (2) to have used BNZ on an exclusive basis; (3) to not have severe Cd; (4) to have conditions for good adherence to the treatment; and (5) to understand and accept the treatment after clarification of the efficacy of BNZ and its adverse events. The treatment exclusion criteria were (1) the use of another trypanosomicide drug alone or simultaneously with BNZ; (2) to have a more severe clinical form of Cd; (3) to have undergone situations that made treatment adherence impossible; and (4) being pregnant.

The study was approved by the Research Ethics Committee of the State University of Campinas in SP, Brazil, through Opinion no. 1266/2011. The patients were informed about all of the steps related to the study, and they signed the Free and Informed Commitment Terms (Termo de Compromisso Livre e Esclarecido). The TCLE was presented to the adult patients after the clarification of potential AEs resulting from the use of BNZ. The patients signed the TCLE freely and willingly. For minor patients, the information was delivered to their parents or legal guardians, who also signed the TCLE freely and willingly. At no time did the Research Ethics Committee/Unicamp waive the requirement of the consent of the parents or legal guardians of the minors.

Indications for the specific treatment with BNZ were those determined and synthesized at the Brazilian Consensus on Chagas Disease [2]. At the time of appointment, the patients were carefully informed about the drug, its efficacy, the duration of treatment, the need to attend non-routine visits for clinical care and the conduct of subsidiary tests, as well as possible AEs. Prior to the drug intake, the patients were submitted to a complete clinical examination, blood count, aspartate aminotransferase (AST), alanine aminotransferase (ALT), alkaline phosphatase, gamma-glutamyltransferase (γGT), bilirubin, albumin, uric acid, creatinine, triglycerides, total cholesterol, LDL and HDL cholesterol fractions and impaired fasting glycemia tests. This routine was repeated at the 30th day, 60th day and end of the treatment. Phone contacts (the secretary and the assistant physician) were provided to the patient; they were free to communicate concerns, especially AEs, emphasizing the cutaneous reactions, digestive disorders, anorexia, weight loss, jaundice and choluria. Additionally, subsidiary tests were important for detecting AEs without regular clinical manifestations: blood dyscrasia, hepatic function and renal function. If these AEs occurred and their severity needed to be assessed, the prescription of antihistamines or corticosteroids was indicated or a previous appointment before the expected time was scheduled for the patient. In the event of the discontinuation of treatment, the subsidiary tests were repeated at the time of this conduct.

The BNZ dose was 5 mg/kg/day for adults and 10 mg/kg/day for children, with doses taken every 8 h, respecting a short interval after meals and aiming to avoid not only fasting but also fatty foods. After the publication of the first Brazilian Consensus on Chagas Disease [13], it was decided that it would not exceed 300 mg/ day but that the period of treatment could be extended to achieve the total dose.

Other data obtained for each patient were age, gender, probable type of transmission, clinical phases and forms of Cd, serological tests after treatment and the occurrence of death.

## Results

**Fig 1:**
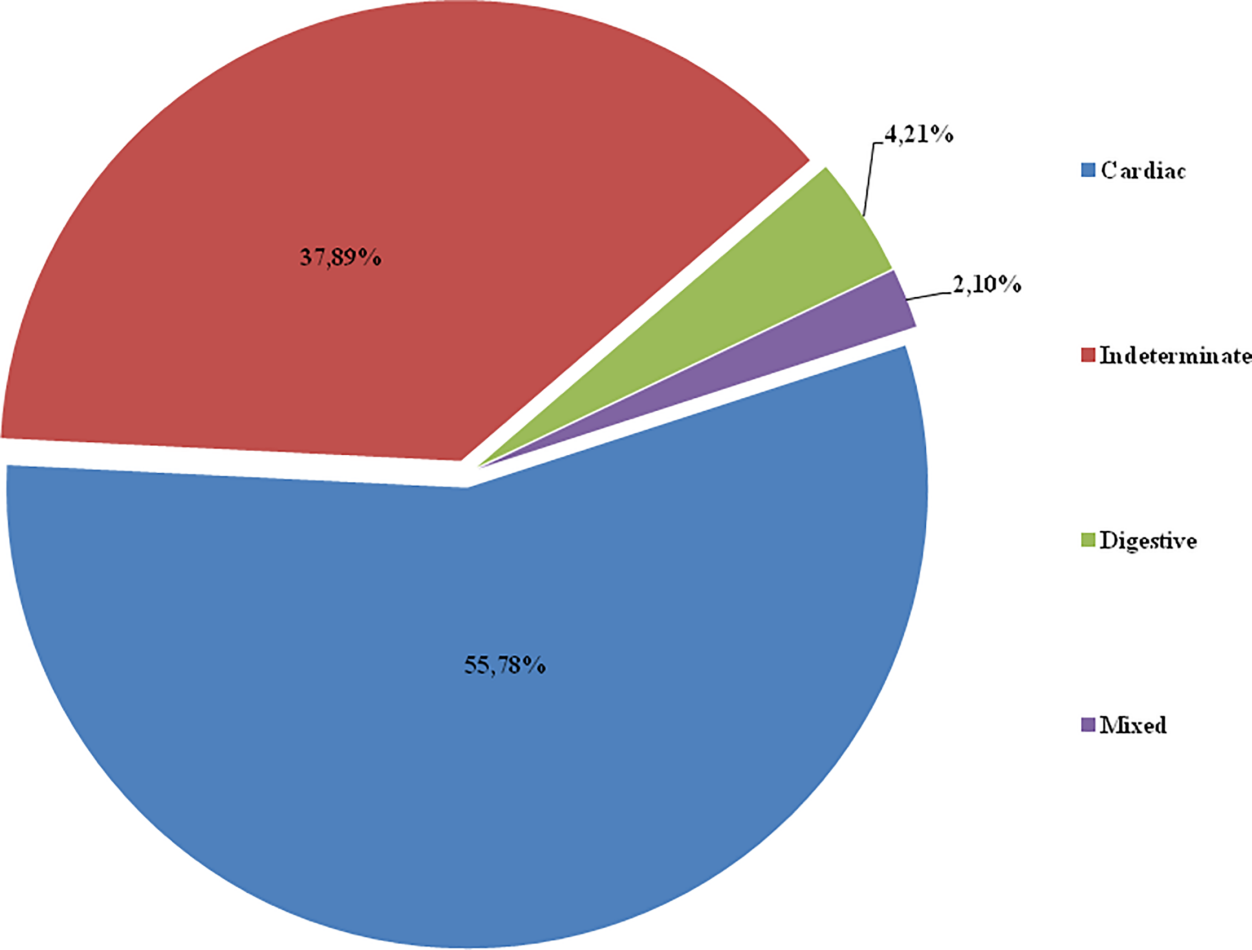
The clinical form. Frequency of clinical forms in patients with chronic dC, who underwent benznidazole treatment.

There were 204 patients who started the treatment with BNZ; 172 (84.31%) completed it. A total of 32 patients (15.68%) discontinued the treatment due to adverse reactions to BNZ. There were 190 (93.13%) individuals in the acute phase of Cd and 14 (6.86%) in its chronic phase. Of the chronic cases, 3 (21.42%) were congenitally transmitted; 3 (21.42%) were transmitted by vector; 4 (28.57%) were acquired due to laboratory accident; and 4 (28.57%) represented exacerbation of chronic Cd in immunosuppressed patients due to antiretroviral drugs upon heart transplantation. The clinical form of the most prevalent chronic phase was cardiac, affecting 106 patients (51.96%). The clinical form was undefined in 72 patients (37.89%), digestive in 8 patients (4.21%), and a mixed form in 4 patients (2.10%). The data are shown in Fig 1.

Adverse events involving cutaneous, hepatic, digestive, hematological and neurological changes related to BNZ were observed in 85 patients (41.66%). Out of these 85 patients, 56 (65.88%) were female, and their ages ranged from 3 years to 66 years (39.60 years ± 12.87 years), with an average of 39 years. Out of the 204 patients who used BNZ, only 128 (62.74%) underwent tests to control liver enzymes, and 35 patients (27.34%) had elevations alone or other associated AEs. These liver AEs were more prevalent in females, who represented 26 of the 35 patients (74.28%). The age range of the patients was 3−66 years (37.53 ± 17.67), with an average of 37 years. The enzymes were elevated in the following order of frequencies: AST, 16 cases (45.71%), with the values ranging from 28 U/L to 220 U/L (74.18 U/L ± 10.60 U/L); ALT, 16 cases, with values ranging from 35 U/L to 310 U/L (99.93 U/L ± 10.60 U/L); alkaline phosphatase, 10 cases (28.57%), with values between 107 U/L and 302 U/L (191.90 U/L ± 6.36 U/L); and γGT, 6 cases (17.14%), with values ranging from 79 U/L to 258 U/L (136.33 U/L ± 3.53 U/L). Table 1 specifies the minimum and maximum observed liver enzyme elevations reached and the maximum number of times that they exceeded the reference value.

**Table 1:** Values of liver enzymes in patients using benznidazole for Chagas’s disease specific treatment.

Liver Enzyme	Minimum Value (U / L)	Maximum Value (U / L)	Maximum Elevation Above Reference Value	Reference Value
**ALT**	35*	310*	9.11 x**	< 50 U / L for men < 34 U / L for women
**AST**	28*	220*	8.18 x**	< 33 U / L for men < 27 U / L for women
**Falc**	107*	302*	2.90 x**	< 129 U / L for men < 104 U / L for women
**γGT**	45*	258*	6.14 x**	< 71 U / L for men < 42 U / L for women

ALT–Alanine aminotransferase. AST–Aspartate aminotransferase. Falc–Alkaline phosphatase γGT–Gamma glutamyltransferase

* Female gender.

x** -Times the Reference Value.

When analyzed separately, the liver enzyme elevations occurred in 17 patients (48.57%), and alkaline phosphatase was the one that was elevated most often, in 10 patients (58.82%), followed by AST and γGT, which each were elevated in 3 patients (17.64%), and finally ALT, which was elevated in only 1 patient (5.88%).

A skin-related adverse event to BNZ occurred in 48 patients (56.47%), of whom 29 were female (60.41%). The minimum age of these patients was 24 years, while the maximum age was 61 years (41 years ± 19.79 years), and the average age was 41 years. The cutaneous reactions had diverse aspects, with morbilliform eruption or erythema only, pruritus, localized or extending throughout the body, and occasionally accompanying edema, fever and lymph node enlargement. The AE was severe and responsible for treatment interruption in 32 patients (66.66%). Its intensity was low or moderate in 16 patients (33.33%). The use of antihistamines or corticosteroids was sufficient to control the dermal reaction in cases of lower severity, allowing for the continuation of treatment until its end.

Epigastric pain was observed in 8 of the patients (9.41%) who used BNZ. The treatment was not suspended for any of the patients, and the pain improved with the use of gastric acid secretion inhibitors, which were used in 4 women of different ages, ranging from 34 years old to 47 years old (40.5 years ± 2.12 years) and having an average age of 42 years.

Hematologic changes, with a decrease in the neutrophil rate, occurred and caused the suspension of the drug treatment in seven patients (8.23%). The blood count normalized rapidly with this procedure without the need for any other intervention or the participation of the hematologist. This AE was more frequent in females, affecting 5 women; the age range of the affected patients was 39−54 years (43.40 years ± 2.82 years), and the average age was 39 years.

**Fig 2:**
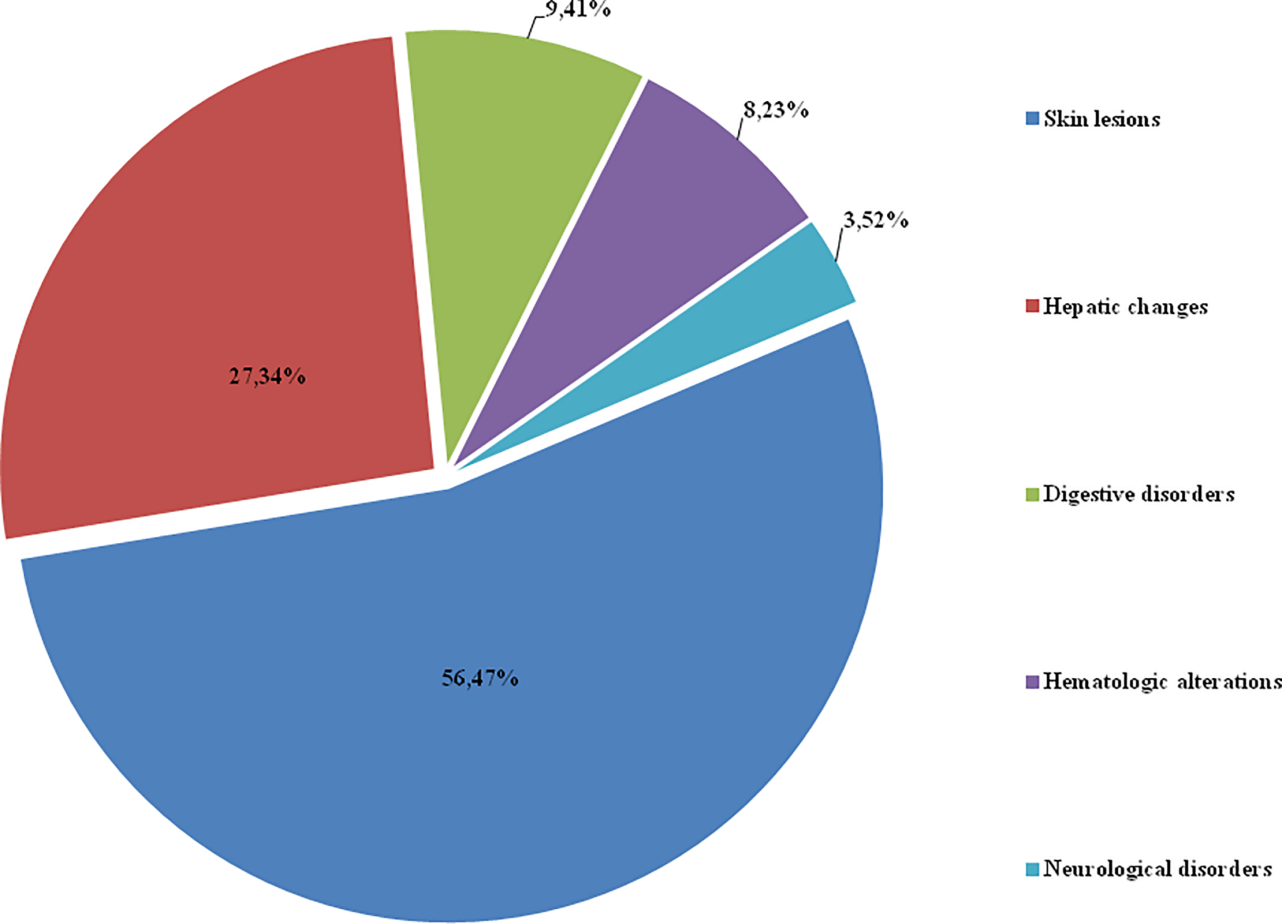
The adverse events. Frequency of adverse events to benznidazole.

Peripheral neuropathy was suspected in 3 (3.52%) patients, who reported a loss of sensitivity, paresthesia or pain in the hands and feet at approximately the 50th day of treatment. Electromyography could be performed in only one patient and did not confirm the diagnosis of peripheral neuropathy but rather radiculopathy. This AE more frequently affected females, with 2 of the patients being women; the minimum age of the affected patients was 25 years, and the maximum age was 33 years (29 years ± 0.70 years). The frequencies of all of the adverse events are shown in Fig 2.

Serological (enzymatic and indirect immunofluorescence) tests were performed at the end of treatment and at annual follow-ups in 101 patients (49.50%). There was a titration decrease in 75 patients (36.76%), no change in 12 patients (5.88%), and fluctuating titers in 6 patients (2.94%). There was no negative serology in any case. There was an increase in the post-treatment serological titers in 8 (3.92%) patients.

Seventeen patients (8.33%) died, all as a result of typical Cd complications that had no relation to the BNZ-specific treatment.

## Discussion

The casuistry of this study, which was conducted with 204 patients who received a specific treatment with BNZ, seems to be less expressive than that of other studies published [14,15] but is similar to and greater than that of many others, as evidenced in a meta-analysis systematic review on the subject [13]. The reference center where the patients in this study were treated followed recent past trends in that the specific treatment did not influence the natural evolution of Cd in its chronic stage. The vector transmission of Cd has been controlled since the 1970s in the region where this center is located, in the state of São Paulo, Brazil, with maximum reduction in the incidence of acute cases and recent chronic stage cases in children and young people [16].

The chronic stage of Cd is usually asymptomatic, and because there are no changes on supplementary tests, patients remain in an undetermined clinical form. However, 30% of patients progress to chronic chagasic cardiopathy over a span of decades and have symptoms including heart failure, arrhythmias and thromboembolism. The observations from longitudinal studies performed in endemic areas with an active vector transmission showed an incidence of 1.85% per year [17,18]. In the current study, the frequency of cardiomyopathy was 51.70%, a value that is higher than what was estimated in the aforementioned studies.

Regarding the liver, the frequency of 27.34% for hepatic changes in this study was observed; among the 204 patients treated with BNZ, enzyme levels were measured in only 128 patients (62.74%) in the follow-up therapeutic procedure. In this regard, the fact that not every patient was assessed can be explained by the heterogeneity of the team responsible for the treatment, as not every member was aware of the need to monitor hepatic changes. Another possible explanation for this is the observation of previous behaviors in the literature, where there is no attention to this issue [18,19,20]. In a few studies reporting the occurrence of liver AEs due to BNZ, the frequency of these changes ranged from 1.8% to 4.9% [6,11]. The results of the current study (27.34%) make it possible to consider liver changes as a frequent AE.

Hepatic changes consisted of elevations of the AST, ALT, alkaline phosphatase and γGT enzyme levels, while the albumin and bilirubin levels remained unchanged. These findings presumably demonstrate that the elevation in liver enzymes does not mean that there is structural damage in the hepatocytes per se. The AST and ALT enzymes are located inside hepatocytes, close to the cell membrane, and depending on the increase in the amount of these enzymes, they may be released from cells, which causes hepatocyte necrosis [21,22]. When a plasma increase in these enzymes reaches 10 or more times the reference values, it can be understood as reflecting hepatitis caused by drugs. In the present study, the values reached a maximum of 9.11 for ALT, 8.18 for AST, 6.14 for γGT and 2.9 for alkaline phosphatase (Table 1). The surveyed literature indicates that a few authors have quantified this elevation (Table 2) and obtained results similar to those observed in this study, with patients presenting digestive symptoms but not suggesting hepatic cell injury. Thus, the elevation of the levels of liver enzymes suggests that the effect of BNZ on the liver generates functional changes in the cholestatic-type hepatocytes that are only detectable in the laboratory in the majority of cases.

**Table 2:** Liver enzymes elevation and presence of other adverse events to benznidazole in literature reports.

Reference	Methods	Sample size (cases)	Follow-up time (years)	AST Elevation	ALT Elevation	Other adverse events
**8**	Review	-	-	3x	3x	Present
**18**	Observational prospective	746	5	> 4x	> 4x	Present
**12**	Prospective	6	2	20 x	20 x	Present
**19**	Prospective	20	5	> 3x	> 3x	Present
**20**	Case report	1	9	> 5x	> 5x	Present

One of the patients drew attention due to a greater elevation of liver enzymes, presenting with digestive symptoms but not featuring the clinical condition of jaundice. The patient also showed a cutaneous reaction and hematological change characterized by neutropenia. All of these changes were rapidly regularized with the suspension of the drug and by treatment with low doses of corticosteroid for a short period of time. The fact that the enzymes returned to the reference values rapidly, as occurred in the patients described in this study and those reviewed in the literature, reinforces the impression that these changes are due to an increase in the amount of the enzymes in the hepatocytes being translated into a functional deficit rather than a necrotic lesion, not aligning drug-induced hepatitis with the nosological concept of the disease. There was a late elevation of liver enzymes at the beginning of the treatment, which was verified in supplementary control tests at the 30th day. Although there is no clear understanding of the mechanisms involved in BNZ-induced hepatic changes, the late elevation of enzymes at the beginning of treatment suggests that there should be a relationship with an increased time of exposure to the drug. Idiosyncratic reaction to the drug seems to be the most likely mechanism responsible for the adverse events caused by BNZ since there are both more than one territory of the affected organism and rapid recovery from the withdrawal of the drug rather than the direct toxic effect of the drug [23,24]. There are no clinical studies in the literature that directly address the mechanisms that involve AEs when benznidazole is used.

However, animal studies were performed with the purpose of elucidating the mechanisms of action of BNZ against *T*. *cruzi* and those responsible for the hepatic AE [25,26,27]. Enzymatic processes mediated by cytochrome P450 generate nitric compounds that result in free radicals from oxygen and nitrogen responsible for toxicity not only to the parasite but also, to a lesser degree, to host cells, culminating in AEs, demonstrated by an elevation of liver enzymes. One experimental study on the mechanism responsible for the effects of BNZ on the liver assessed structural lesions in the organ and its collagen content [27] by histopathological analysis [25]. The findings indicated signs of liver hypertrophy in the group of animals using BNZ but did not detect an increase in collagen. The authors believe that there are few standardized structural, ultrastructural and biometric studies on the models investigated for definitive conclusions on the subject. However, the lack of collagen enhancement may be understood as indicating that the replacement of hepatic parenchyma by cicatricial connective tissue is not required, suggesting that there is no necrosis produced by BNZ.

It is noteworthy that the patient who presented with a higher enzyme elevation used concomitant statin treatment for the treatment of hypercholesterolemia. Because statins use the cytochrome P450 metabolic pathway, they may interact with other drugs that use the same metabolic pathway [12,25]. Statins act primarily in the liver, where a special transport system allows for their incorporation into the hepatic tissue for biotransformation [26]. Thus, it cannot be excluded that the BNZ interaction with statins may be responsible for the greater severity of the reported case. In a letter to the editor [27], the authors reported a case that presented jaundice as early as the third day of treatment with BNZ. They pointed out the lack of similar observations by professionals who repeatedly, and for a long time, prescribed BNZ. However, the patient had an expressive indirect bilirubin elevation. Concomitantly, the patient had glucose-6-phosphate dehydrogenase deficiency (G6PD), and the coincidence with hemolytic crisis, and also an interaction with BNZ, cannot be excluded. A similar situation was described in a summary presented at a scientific event in which the authors reported an individual with Cd who had direct (1.6 mg/dl) and indirect (4.6 mg/dl) bilirubin elevation with regular AST and ALT levels, besides anemia, after 28 days of BNZ use [28]. Studies with large numbers of patients informing hepatic AE report no jaundice, no hyperbilirubinemia and no hemolysis dependent on this drug. Thus, it is difficult to attribute the elevation of bilirubin to BNZ in the cases described. However, they were the only reports found in the surveyed literature that presented such findings in isolated cases. No studies on which drugs may interact with BNZ or detailed information about drugs used concomitantly with BNZ have been found. Thus, the possibility of drug interactions, or other liver diseases, and the triggering of BNZ-dependent hepatic changes remain speculations and suggestions for further investigation.

Another situation raised by an experimental study on rats stated that there could be interaction between the hepatic metabolism of BNZ and infection by *T*. *cruzi* since liver involvement may occur in the natural course of Cd and both situations require a hepatic response for the purpose of neutralizing nitro and oxidative stress [25]. This experimental study assessed animals in the acute stage of the disease in which hepatic changes can occur since it is a systemic disease. The description of hepatosplenomegaly in this situation contributes to this impression [4]. However, it cannot be said that it occurs in the chronic stage of Cd, although the parasite can be found in the liver and in other organs. The same experimental study concluded that the group of animals taking BNZ had greater liver abnormalities than the control group with chagasic infection alone and that the relation of the two situations was not different, concluding that hepatic changes by BNZ occur independently of the stage of the disease. Finally, for 48.57% of individuals who had an elevation of liver enzymes, the elevation occurred independently from other AEs. This demonstrates the need to assess the liver during treatment with BNZ, without which this AE would go unnoticed and the drug use would be continued.

Because BNZ pharmacokinetic studies in children show a lower plasma concentration of the drug [29,30] and a lower frequency of the clinical signs of AEs, treatment with lower doses and different dosing regimens are often used in adults. This is because higher BNZ plasma concentrations would be related to a higher frequency of AEs. A new dosing regimen for BNZ treatment was prepared by administering the same dose of 5 mg/kg/day at five-day intervals for 60 days [31,32]. However, the AEs, including the hepatic AEs, typical under standard dosages were not changed with this new therapeutic pattern, in regards to both strength and quantity. This demonstrates that the mechanisms responsible for the AEs to BNZ are not yet completely elucidated.

The efficacy of BNZ in the treatment of chronic Cd in the patients of the current study should be the objective of another publication, given the relevance of the topic. However, illustratively, there was no negative serology in any case.

The data observed in this study allow us to conclude that adverse events related to the liver from BNZ occur and are frequent but are controllable and not severe. They represent elevations of liver enzymes and should be monitored mainly at the 30th day and at the end of the treatment.

## Supporting information

S1 Checklist STROBE checklist. (DOCX)
