# Supplementary material for: Hepatic changes by benznidazole in a specific treatment for Chagas disease
Source: PLoS One. 2018 Jul 20;13(7):e0200707. doi: 10.1371/journal.pone.0200707 (PMC6054377; doi:10.1371/journal.pone.0200707)
Supplement: S1 Checklist — (DOCX) [file pone.0200707.s001.docx]

***S1 Checklist: STROBE Checklist***

STROBE Statement—Checklist of items that should be included in reports of ***cross-sectional studies***

|  | Item No | Recommendation |
| --- | --- | --- |
| **Title and abstract** | 1 | (*a*) Transverse cohort study to report the adverse events due to the use of benznidazole in a specific treatment for Chagas’ disease, with a main focus on hepatic changes. |
|  |  | (*b*) Of the 204 patients who used benznidazole, 85 cases (41.66%) with adverse drug events and 35 (41.17%) of them related to the liver, characterized by elevation of AST liver enzymes, ALT, alkaline phosphatase and gamma-glutamyltransferase. |
| Introduction | | |
| Background/rationale | 2 | It is known that benznidazole causes adverse events in adult chagasic patients, such as the high frequency of cutaneous reaction, which is much reported in the literature. There is little experimental and human study on the adverse event of benznidazole on the liver, but the few are categorical in stating that benznidazole causes elevation of liver enzymes without impairing organ function. |
| Objectives | 3 | To verify the frequency of adverse events due to the use of benznidazole in chagasic patients and if in some of these patients the elevations of liver enzymes such as AST, ALT, alkaline phosphatase and gamma-glutamyltransferase were observed. |
| Methods | | |
| Study design | 4 | Chagas disease; Benznidazole; Epidemiology; Adverse events; Treatment; *Trypanosoma cruzi* |
| Setting | 5 | The study was carried out at the outpatient clinic of the Chagas Study Group (GEDoCH), belonging to the State University of Campinas / Brazil. All medical records included 204 patients who used benznidazole between 1979 and 2016. Serological, haematological, hepatic and urinary exams were performed prior to initiation of treatment and these examinations were repeated during treatment and at the end. Patients are followed up indefinitely. |
| Participants | 6 | (*a*) To analyze whether patients who used benznidazole presented clinical or laboratory adverse events by reading medical records. |
| Variables | 7 | The 204 patients assessed were 40.6 ± 13.5 years and 104 were women (50.98%). Fourteen (6.86%) individuals were found to be in the acute phase of Chagas’ disease, and 190 (93.13%) in its chronic phase. Adverse events occurred in 85 cases (41.66%), being 35 (41.17%) of them related to the liver, characterized by an elevation of AST liver enzymes, ALT, alkaline phosphatase and gamma-glutamyltransferase. Other AEs observed were: cutaneous changes in 48 cases (56.47%), 8 cases of epigastric pain (9.41%), 7 cases of blood dyscrasia (8.23%), and 3 cases of peripheral neuropathy (3.52 %). Treatment was interrupted in 32 cases (37.64%) due to adverse events. |
| Data sources/ measurement | 8* | Chagasic patients treated with benznidazole. Collect epidemiological data as place of birth, clinical form of disease, age at treatment time. Analyze the blood tests before the treatment and its possible variations during the treatment. Identify if there were adverse events due to the drug, including hepatic changes. |
| Bias | 9 | Through the medical records of each patient treated with benznidazole the frequency of clinical and laboratory adverse events can be verified. |
| Study size | 10 | For the medical records, the number of 204 patients who used the medication was found. And through this survey he observed the frequencies of each clinical and laboratory adverse events. |
| Quantitative variables | 11 | Clinical and laboratory adverse events were calculated by the mean and standard deviation and median. |
| Statistical methods | 12 | (*a*) A statistical analysis was simple, not requiring the use of statistical tests. It analyzed the mean arithmetic, standard deviation and median of the data collected |
|  |  | (*b*) We sought data from the patients' medical records. |
|  |  | (*c*) All the data proposed for analysis were found. |
|  |  | (*d*) No analytical method was applicable. |
|  |  | (*e*) The sensitivity test was not done. |
| Results | | |
| Participants | 13* | (a) Approximately 4000 patients were seen in the outpatient clinic since 1979. Of these patients, 204 used benznidazole and only 35 patients had adverse events with hepatic enzymatic alterations. |
|  |  | (b) Only 204 patients were selected because they received benznidazole to combat the parasite Trypanosoma *cruzi*. |
|  |  | (c) There was no need to use diagram. |
| Descriptive data | 14* | (a) Chagas' patients aged 3 years to 66 years used benznidazole. The study analyzed the age, place of birth, clinical form of the disease and clinical and laboratory adverse events of each patient.. |
|  |  | (b) Did not find blood results from 126 patients. |
| Outcome data | 15* | Adverse events occurred in 85 cases (41.66%), being 35 (41.17%) of them related to the liver, characterized by an elevation of AST liver enzymes, ALT, alkaline phosphatase and gamma-glutamyltransferase. Other adverse events observed were: cutaneous changes in 48 cases (56.47%), 8 cases of epigastric pain (9.41%), 7 cases of blood dyscrasia (8.23%), and 3 cases of peripheral neuropathy (3.52 %). Treatment was interrupted in 32 cases (37.64%) due to adverse events. |
| Main results | 16 | (*a*) There were no adjusted estimates. |
|  |  | (*b*) There were no category limits. |
|  |  | (*c*) It will not be relevant. |
| Other analyses | 17 | There were no other analyzes done. |
| Discussion | | |
| Key results | 18 | As expected from the literature reports, adverse events were found, such as mild to severe skin reactions. Severe skin reactions resulted in discontinuation of treatment. Adverse events such as haematological changes were found, more specifically the reduction of leukocytes. The frequency of hepatic and neurological changes were also found at a lower frequency. Thus, the need to perform examinations before the beginning and during the treatment is reinforced. |
| Limitations | 19 | The limitation was that not all patients who used benznidazole had blood tests before and during treatment. Thus, we do not have the actual frequency of hepatic changes in the patients treated at the outpatient clinic in question. There were few reports of cases due to hepatic alteration due to the use of the drug. We believe that further studies are necessary for the actual perception of this adverse event. |
| Interpretation | 20 | This study reinforces the need to perform haematological and hepatic exams prior to initiation of benznidazole treatment as a control of possible metabolic abnormalities. Regarding the neuropathy adverse event that appears at the end of treatment, it is recommended to perform the pre-treatment electromyography examination, which may often be under diagnosed. Regarding the literature, adverse events such as cutaneous and haematological alterations showed an expected correlation.. |
| Generalisability | 21 | We infer that some reference centers have never presented cases of hepatic alterations and may be because they do not perform hepatic exams before the beginning of treatment, because in our study we noticed some flaws about this. |
| Other information | | |
| Funding | 22 | There was no funding to develop this study. |

*Give information separately for exposed and unexposed groups.

**Note:** An Explanation and Elaboration article discusses each checklist item and gives methodological background and published examples of transparent reporting. The STROBE checklist is best used in conjunction with this article (freely available on the Web sites of PLoS Medicine at http://www.plosmedicine.org/, Annals of Internal Medicine at http://www.annals.org/, and Epidemiology at http://www.epidem.com/). Information on the STROBE Initiative is available at www.strobe-statement.org.
